# Ricin intoxication by lethal dose of castor seeds ingestion: a case report

**DOI:** 10.1186/s13256-024-04697-8

**Published:** 2024-08-30

**Authors:** Lysette Joelle Noumi Noumi, Sylvana El-Hanna, N. M. Reine Sandrine Mendeuka, Marc Van Nuffelen

**Affiliations:** 1 Department of Emergency-Medicine, Saint Elisabeth Clinic, Namur, Belgium; 2 Department of Emergency-Medicine, Lebanese University, Faculty of Medical Sciences, Mount Lebanon, Lebanon; 3 Department of Emergency-Medicine, Hospital of Mons Groupe-Jolimont, Mons, Belgium; 4 Department of Emergency-Medicine, Saint Luc University Clinics, Brussels, Belgium

**Keywords:** Castor seeds, Ricinus communis, Ricin, Intoxication

## Abstract

**Introduction:**

Ricin intoxication is a serious condition with symptoms ranging from mild gastroenteritis to fatal outcomes due to shock and multi-organ failure. Intoxication from the ingestion of castor seeds is uncommon. However, its diagnosis is crucial, particularly with a clear history of exposure to castor seeds, regardless of the route of exposure (enteral or parenteral). Prompt diagnosis is essential to monitor and manage the patient effectively and to prevent potentially fatal outcomes. We report a case where ingestion of castor seeds resulted in gastroenteritis severe enough to necessitate emergency medical care.

**Case report:**

We present the case of a 47-year-old Belgian woman of Moroccan descent, previously healthy who was admitted to the emergency department with symptoms of colicky abdominal pain, diarrhea, and vomiting following the ingestion of six castor beans. The patient was diagnosed with ricin intoxication, admitted for observation, and received symptomatic treatment. She was discharged home after a complete recovery three days later.

**Conclusion:**

Our report underscores the clinical manifestations, hemodynamic changes, laboratory findings, and treatment of intoxication due to castor seed ingestion. It contributes to the limited literature on castor seed poisoning in humans, with a specific focus on cases in Belgium. This report aims to raise awareness among clinicians about this condition and emphasizes the importance of a comprehensive history-taking to prevent misdiagnosis and malpractice.

## Introduction

Ricinus communis, commonly known as the castor oil plant [1], belongs to the Euphorbiaceae family. It is an herbaceous annual crop cultivated globally, primarily for extracting castor oil, which has applications in both pharmaceutical and industrial sectors [2]. Industrially, the demand for castor oil is increasing, driven by incentives for biodiesel production [2], and its use as a lubricant, coating agent, component in plastic products, and as a fungicide [2]. Pharmacologically, castor oil is recognized for its cathartic, purgative, antidiabetic, antifungal, antibacterial, analgesic, and antinociceptive properties [3]. Post oil extraction, a by-product known as castor cake, rich in protein and fiber, is produced [2]. However, its usage is restricted due to the presence of toxic compounds, primarily Ricin.

Ricin toxicity can occur through ingestion, inhalation, or injection. No cases of poisoning from purified ricin have been documented in the literature, with all clinical reports of poisoning being associated with castor bean ingestion. Previous studies suggest the lethal oral dose of ricin for humans ranges from 1 to 20 mg/kg of body weight [4], although there is considerable variability in clinical response to castor bean ingestion, with reports indicating that consuming 0.5–30 beans can trigger symptoms [1].

This brings us to our case, where the patient ingested six castor beans. This situation highlights the potential risks associated with castor seeds, a well-known herbal product that can cause a range of symptoms, from mild to severe, depending on factors like the route of exposure, type of seeds, the patient’s medical history, and age. In our case, the patient presented with symptoms akin to gastroenteritis, underscoring the need to raise awareness among clinicians and the general population about this relatively uncommon but potentially severe medical condition.

## Case report

A 47-year-old Belgian woman of Moroccan descent, with no significant medical or psychiatric history except for a cesarean section in 2006, presented to the emergency department on November 9, 2022, at 12:16 pm. She was previously healthy, a nonsmoker, abstained from alcohol, and had no known food or drug allergies.

The patient reported diffuse, fluctuating, and cramping abdominal pain of several hours' duration, without radiation or identifiable exacerbating or alleviating factors. This pain was associated with non-bilious vomiting and succeeded by multiple episodes of non-bloody, watery diarrhea. She denied experiencing fever, chills, cardiac symptoms, respiratory issues, significant neurological symptoms beyond a mild headache, or genitourinary complaints.

Upon evaluation, her vital signs were recorded as follows: blood pressure 109/65 mmHg, pulse 80 bpm, temperature 36.9 °C, and oxygen saturation 98%. Physical examination revealed normal bowel sounds and diffuse abdominal tenderness without rebound or guarding. There was no costo-vertebral angle tenderness or suprapubic pain. Cardio-respiratory examination showed normal heart sounds without murmurs and clear lung fields without adventitious sounds. Her carotid pulses were normal bilaterally with no bruits. Neurologically, she was alert, cooperative, and oriented, with no deficits, cerebellar or meningeal signs; her pupils were equal and reactive to light, and no lymphadenopathy was palpable.

Upon presentation to the emergency room (ER), the patient's laboratory tests were largely within normal limits, with white blood cells (WBC), red blood cells (RBC), hemoglobin, and blood urea nitrogen (BUN) nearing the upper normal range. Notably, there was neutrophilia accompanied by lymphopenia, while C-reactive protein (CRP) levels remained normal and no renal or hepatic function disturbances were observed (Table 1). Additional tests, including urine analysis, blood culture, stool analysis and culture, and a SARS-CoV-2 PCR, were conducted.

**Table 1:** Laboratory results on admissions and on the following days

#	Parameter	Reference range	9/11/2022 13:29	9/11/2022 19:07	10/11/2022 04:10	11/11/2022 06:45
1	APTT Actin FS (s)	21.6–28.7	20.2	22.2	22.8	21.8
2	Actin FS ratio	0.80–1.20	0.86	0.94	0.97	0.93
3	PT (%)	70–130	109	99	99	86
4	INR	0.95–1.31	0.96	1	1	1.06
5	PT (s)	9.8–12.5	10.5	10.9	10.9	11.5
6	WBC × 10^3^/mm^3^	3.5–11	10.21	8.76	9.36	
7	RBC × 10^6^/mm^3^	3.8–5	5.36	4.55	4.60	
8	Hemoglobin (g/dl)	12–16	15.4	13.2	13.2	
9	Hematocrit (%)	35–47	46.8	39.3	39.3	
10	MCV (μm^3^)	80–100	87.3	86.4	85.4	
11	MCH (pg)	26–34	28.7	29	28.7	
12	MCHC (g/dl)	31–36	32.9	33.6	33.6	
13	RDW (%)	11.5–13.4	12.6	12.8	12.8	
14	Platelets × 10^3^/mm^3^	150–440	309	253	226	
15	MPV (μm^3^)	6.8–10	11.3	11.4	11.1	
16	Neutrophils (%)	40–75	92			
17	Absolute neutrophils × 10^3^/mm^3^	1.5–6.7	9.4			
18	Lymphocytes (%)	20–40	6.5			
19	Absolute lymphocytes × 10^3^/mm^3^	1.2–3.5	0.66			
20	Monocytes (%)	2–8	1.1			
21	Absolute monocytes × 10^3^/mm^3^	0.1–1	0.11			
22	Eosinophils (%)	1–4	0			
23	Absolute eosinophils × 10^3^/mm^3^	0.1–0.5	0			
24	Basophils (%)	0–1	0.4			
25	Absolute basophils × 10^3^/mm^3^	0.00–0.15	0.04			
26	CRP (mg/l)	< 5	1.1	1.3		
27	BUN (mg/dl)	16.6–48.5	36.8	30.6	18.1	8.8
28	Creatinin (mg/dl)	0.50–0.90	0.89	0.74	0.58	0.60
29	GFR ml/min/1.73 m_2_	> 60	77	96	110	108
30	Sodium (mmol/l)	136–145	141	137	138	141
31	Potassium (mmol/l)	3.4–4.4	3.8	3.6	3.8	3.7
32	Chlore (mmol/l)	98–107	103	102	104	105
33	CO_2_ (mmo/l)	23–29		22	22	25
34	Total bilirubin (mg/dl)	< 1.2	0.40	0.36	0.52	0.30
35	Direct bilirubin (mg/dl)	< 0.20		0.14	0.18	
36	Alkaline phosphatase (U/l)	35–104	89			65
37	GGT (U/l)	6–42	14			9
38	SGPT (U/l)	< 33	22	14	17	18
39	SGOT (U/l)	< 32	24	22	25	24
40	LDH (U/l)	135–214	216	180	160	172
41	CK (U/l)	26–192	152	132	138	
42	FBS (mg/dl)	70–100	122			
43	Calcium (mmol/l)	2.15–2.50		2.20		2.23
44	Magnesium (mmol/l)	0.63–1.05		0.79		0.82
45	Phosphorus (mmol/l)	0.75–1.39		1.04		0.83

The patient's clinical presentation of abdominal pain and vomiting, along with a history of cesarean section, initially broadened the differential diagnosis to include peptic ulcer disease, biliary or renal colic, and intestinal occlusion. However, the acute onset of watery diarrhea, exceeding ten episodes from symptoms onset to ER visit, coupled with the initial symptoms of vomiting and abdominal pain, her physical examination, vital signs, and laboratory findings, suggested a probable diagnosis of self-limiting viral gastroenteritis including SARS-CoV-2 infection but a negative SARS-CoV-2 PCR excluded this latter as an initial cause. Symptomatic treatment with Scopolamine Butylbromide 20 mg IV for abdominal cramps, Alizapride 50 mg IV for vomiting, and intravenous rehydration with 0.9% NaCl provided partial symptom relief.

Before discharging the patient on symptomatic treatment, with instructions for follow-up with her primary physician, she disclosed ingesting six ricin beans on November 8, 2022, at 10 pm, aiming to prevent hair loss and enhance hair thickness and health. She was influenced by a natural herb store vendor, who touted the benefits of ricin seeds for hair loss treatment and prevention and demonstrated their consumption. The patient began experiencing symptoms ~ 4–5 h after ingestion.

Her history of castor seed ingestion and the associated clinical manifestations were reported to the Poison Control Center in Brussels, which acknowledged the connection between the ricin seed ingestion and her symptoms. They highlighted potential complications, such as dehydration, gastrointestinal bleeding, hypovolemic shock, and multi-organ failure, recommending ICU monitoring due to the potentially fatal quantity of ingested seeds and the risk of clinical deterioration. They also advised measuring ricinine levels in urine and plasma for documentation, although these tests were unavailable at our hospital and nearby facilities.

Consequently, the patient was admitted to the ICU for close observation. Her laboratory tests, conducted twice at nine-hour intervals, remained within normal ranges, showing no increase in CRP or leukocytes, no anemia, and no liver or renal function abnormalities (Table 1). Her vital signs stayed normal and stable throughout the ICU stay.

The patient received isotonic saline (0.9% NaCl) for maintenance hydration and was administered symptomatic pharmacotherapy, including Alizapride 50 mg IV for antiemetic purposes and Scopolamine 20 mg IV as needed. Additionally, Pantoprazole 40 mg IV was administered as a proton pump inhibitor (PPI). Her primary symptoms of vomiting, diarrhea, and abdominal pain resolved spontaneously without the emergence of new symptoms or complications during her 24-h stay in the Intensive Care Unit (ICU). Following this, she was transitioned to the internal medicine ward for continued observation. Over the subsequent 48 h, her treatment regimen was maintained, and repeat laboratory evaluations remained within normal parameters (Table 1). Two sets of blood cultures, as well as stool and urine analyses, yielded negative results.

After a total hospital stay exceeding 72 h, the patient was discharged, having achieved complete symptomatic resolution and without any hemodynamic instability or laboratory abnormalities. She was advised to seek immediate medical attention in the event of recurrent gastrointestinal symptoms, the onset of new symptoms such as melena or rectal bleeding, or any other non-gastrointestinal manifestations. A follow-up with her primary care physician was recommended one week post-discharge for further clinical and laboratory evaluation, contingent upon the persistence of an asymptomatic status.

## Discussion

This case involves a 47-year-old patient who presented to the emergency room (ER) with gastroenteritis resulting from ricin toxicity due to oral ingestion of ricin seeds. The onset of symptoms occurred 5 h post-ingestion, with the patient seeking ER care after 14 h. Although the specific ricinine level tests in urine and plasma were unavailable at our facility to confirm the diagnosis, it was established based on a clear history of exposure and typical clinical presentations of gastroenteritis-like symptoms. The differential diagnosis initially favored other causes of diarrhea, but these were deemed less likely given the patient's non-travel history outside Belgium, absence of consumption of food or beverages from unknown sources, lack of contact with ill individuals, and no exposure to medications or toxic substances. Initially, a self-limiting viral gastroenteritis, specifically due to SARS-CoV-2, was considered, especially since gastrointestinal manifestations of SARS-CoV-2 infection were commonly observed in the ER. However, the diagnosis shifted after the patient disclosed her ingestion of castor beans.

Upon admission, the patient underwent a comprehensive series of laboratory tests to monitor her transaminases, renal function, electrolytes, coagulation parameters, and hemoglobin. This approach was crucial due to ricin’s potential to cause circulatory collapse, shock, and multiorgan failure. Accordingly a fluid replacement therapy by NACL 0.9% was started in ER and maintained during her hospital stay along with a symptomatic treatment for her crampy abdominal pain and vomiting.

Ricin is extracted from Ricinus communis L., belonging to the Euphorbiaceae family, and commonly referred to as the castor bean or palma Christi. Ricinus is a monotypic genus, with the castor oil plant as its sole species. The plant likely originated in Africa and Asia and has since proliferated across temperate, subtropical, and tropical regions, either as an invasive species or through cultivation for various uses [6]. Historically, it has been integral to the traditional medical practices of Mediterranean and Eastern ancient cultures [6]. Globally, the castor bean has found diverse applications in folk medicine. In the Mediterranean regions of Europe, it has been used as a galactagogue, where fresh leaves or their juice are applied to the breast to stimulate lactation. In Africa, the plant has been employed to treat joint, skin, and eye ailments, with crushed seeds or oil, often combined with other botanicals, applied topically or ingested to induce uterine contractions for abortive purposes. In the Caribbean, it has been utilized to treat erysipelas, flu, uterine and abdominal inflammations, often in the form of a leaf poultice. In Brazil, castor seed oil is ingested or applied locally as an anthelmintic, purgative, anti-alopecia agent, and to treat wounds or burns [6]. The laxative and anthelmintic effects of castor seeds are partly attributed to the irritative action of ricin on the intestines [6].

Ricin has garnered significant scientific interest for its potential as an immunotherapeutic agent in cancer treatment due to its ability to inhibit protein synthesis. Its lethal properties have also led to its exploration as a military weapon, evidenced by the development of a "ricin bomb" by the British military during World War II. Moreover, the potential misuse of ricin as a bioterrorism agent [3] has elicited serious security concerns.

Ricin is found in concentrations of 1–5% in castor bean seeds. It is water-soluble and, therefore, not expected to be present in castor bean oil. Purified ricin is a white powder, soluble in water, and stable under ambient conditions. It can be inactivated by heating for 10 min at 80 °C or for one hour at 50 °C at a pH of 7.8 [7–9]. Ricin is a glycoprotein lectin, consisting of two chains, A and B, connected by a disulfide bond. The B chain facilitates binding to cell surface receptors and the entry of the toxin into cells. The A chain disrupts the degradation pathway for misfolded proteins in the endoplasmic reticulum, and then, once relocated into the cytosol, it inhibits protein synthesis by removing an adenine from the sarcin-ricin loop of the 28S rRNA of ribosomes [6–9]. In addition to inhibiting protein synthesis, ricin has been linked to other cytotoxic mechanisms, including electrolyte imbalance, cellular membrane damage or structural alterations, the release of inflammatory cytokines, hepatic oxidative stress, and direct induction of apoptosis [1, 9].

Ricin is among the most potent plant toxins. The severity and characteristics of ricin toxicity or poisoning significantly vary based on the dosage and route of exposure (oral, inhalation, injection, or dermal), as well as individual factors such as the extent of mastication, age, and comorbidities [9].

The estimated lethal dose of ricin by inhalation or injection is ~ 5–10 μg/kg body weight in humans, while the lethal oral dose is estimated to be around 1–20 mg/kg body weight, equivalent to roughly 5–8 beans. Notably, the toxicity for intravenous, inhalation, and intraperitoneal routes is ~ 1000 times higher than that for the oral route [5, 6, 9]. Clinical reports of ricin ingestion have documented a range of symptoms, from mild to lethal, with ingested quantities varying from half to thirty beans, and the minimum number associated with fatalities being two [4].

Inhalation of ricin leads to a gradual onset of respiratory distress, characterized by difficulty breathing, coughing, fever, pulmonary lesions, and edema. Death results from respiratory failure due to extensive alveolar fluid accumulation [6].

Parenteral administration of ricin causes immediate local pain at the injection site, followed by general weakness within 5 h. Subsequent symptoms, which are general and may resemble sepsis (including fever, headache, dizziness, anorexia, nausea, vomiting, hypotension, abdominal pain), can be delayed for up to 10–12 h, even with high doses. The condition may progress to multisystem organ failure [6].

Whole ingested beans can pass through the gastrointestinal tract intact, but chewing facilitates the release of ricin [6]. The onset of symptoms after ingestion typically occurs within 4–6 h, but can be delayed up to 10 h [4]. Initial symptoms are nonspecific, including colicky abdominal pain, vomiting, diarrhea, heart burn, and oropharyngeal pain. Less commonly reported are hematemesis and melena. Fluid loss can lead to electrolyte imbalances, dehydration, hypotension, and circulatory collapse [1, 3–8]. Laboratory findings may show leukocytosis, elevated transaminases and creatinine kinase, hyperbilirubinemia, renal insufficiency, and anemia [4, 8]. Depending on the ingested dose, death may occur within 2–5 days [5].

In the literature review, Staňková et al. documented a case of 25 years old patient who died from multiorgan failure six days after an intravenous injection of castor bean extract in a suicide attempt [5]. Conversely, Bucaretchi et al. reported on a 21-year-old man who survived after injecting castor bean extract intramuscularly and subcutaneously, which led to multiorgan failure and necrotizing fasciitis, requiring extensive treatment including fluids, pressors, fasciotomy, debridement, antibiotics, and skin grafts [10]. Wang et al. described seven cases of children with gastrointestinal, neurological, and cardiac symptoms following castor seed ingestion, all of whom improved after plasma exchange and were discharged asymptomatic, highlighting the potential benefit of plasma exchange in treating castor seed toxicity [1]. Thornton et al. analyzed 84 patients who ingested a median of 10 castor seeds and primarily exhibited gastrointestinal symptoms; these patients were treated symptomatically with minimal morbidity and no mortality reported [8]; Kopferschmitt et al. recounted a 21-year-old student who ingested 30 castor beans, few of which were masticated, in a suicide attempt. The student presented to the ER with severe gastrointestinal symptoms, extracellular dehydration, and circulatory collapse but recovered following symptomatic treatment and the infusion of saline and dextrose [11]. Lopez et al. described a teenager who ingested 200 castor beans mixed with juice in a suicidal attempt and presented with neurological and gastrointestinal symptoms (lightheadedness, weakness, nausea and vomiting), recovered after symptomatic treatment and later being transferred to psychiatric ward [12]. Additionally, Abbes et al., in their review of ricin poisoning, found 50 cases worldwide, most presenting with gastroenteritis-like symptoms post-ingestion. These cases were treated symptomatically with fluid repletion, and digestive decontamination using activated charcoal and/or gastric lavage within a day after ingestion to minimize gastrointestinal absorption of ricin, proving early decontamination to be effective [13].

The management of ricin toxicity primarily involves supportive care, as there is no available antidote, vaccine, or specific treatment for ricin poisoning or prevention. Supportive care remains the cornerstone for mitigating morbidity and mortality associated with ricin exposure [1, 4, 8, 11–14]. Research efforts are underway to develop treatments or preventive measures using neutralizing antibodies against ricin, though comprehensive clinical studies are yet to be conducted [14, 15].

A critical insight from the literature review on ricin intoxication is the unpredictability of its prognosis. While some patients succumb to intoxication via ingestion or injection, others do not. Despite its well-known toxic potential, documented cases of human toxicity due to ricin are rare. This case has been reported to contribute to the existing and evolving literature on ricin toxicity and its clinical manifestations, highlighting the severe risks associated with the ingestion of castor seeds. Our patient, who ingested six castor beans—a quantity potentially lethal according to some studies—exhibited mild gastroenteritis symptoms, was managed with supportive care, and was discharged after a full recovery three days later. However, it is crucial to recognize that a similar ingestion could result in near-fatal or fatal outcomes in other individuals.

## Conclusion

Gastroenteritis, characterized by abdominal pain, vomiting, and diarrhea, has a broad differential diagnosis, encompassing both infectious and non-infectious causes, such as exposure to chemicals or toxins. However, castor seed ingestion, a potentially fatal form of poisoning, should be considered, particularly when the patient's history suggests recent exposure. This report seeks to heighten awareness among clinicians and the general public about the risks associated with this toxin contained in a well-known herbal product that is used frequently in an oil texture for cosmetic reasons, therefore it could be easily misused whether intentionally or incidentally.

It emphasizes the critical role of thorough history taking in establishing an accurate diagnosis, thereby guiding appropriate management and preventing malpractice.

## Data Availability

All relevant data are within the paper.
